# The Mystery of Influenza

**Published:** 1893-09-02

**Authors:** 


### The Hospital

An Institutional Journal of

Science, Medicine, Nursing, and Philanthropy.

*For Hospitals, Asylums, Infirmaries, Dispensaries, Medical Practitioners, Students, Nurses, and the Charitable Public.*

Vol. XIV.—No. 362.] [Sept. 2, 1893.

### THE MYSTERY OF INFLUENZA.

The report which has just been laid before the President of the Local Government Board on the recent epidemics of influenza is an interesting but most inconclusive document. Influenza had been for more than a generation a matter of history, or rather of tradition, and the name had been given to every cold in the head that seemed a little more severe than usual, when the genuine disease reappeared, to be received first with incredulity, then with fear. The scientists went promptly to work to investigate the new disease, and so far as the pathology went succeeded admirably. There seems to be no reason for doubting Dr. Klein's conclusion, that influenza is caused by the presence of a micro-organism, which flourishes in the mucous membrane of the respiratory tract, but is found only rarely and, as it were, by accident in the blood. The bronchial territory is its home, and there it works its mischief. It seems evident also that influenza is a highly infectious disease, and that the secretions of the mouth play a highly important part in conveying the poison-bearing bacillus from one person to another.

The popular impression was, of course, that it was atmospheric—"in the air," as people say. That opinion has been held regarding every epidemic that ever appeared, and is a most convenient theory for the indifferent and the lazy, who never go on to ask the apparently obvious question—how did it get into the air and how can it be got out? That cases occurred at the Caskets Lighthouse, which is situated on a rock in the Channel Islands remote from the land, seemed a confirmation of the atmospheric theory. Two keepers were attacked on January 10th, 1892, and two more on January 16th. But the disease had been common in Guernsey, and not unknown in Alderney, and the last communication which the keepers had had with the shore was on the first day of the year. The incubation period in influenza rarely lasts as long as nine days; but the period cannot be considered too long to preclude the possibility of direct infection. This instance is, at best, inconclusive. So is one reported from Northam, Devon, though after another fashion. Seven persons drove to Northam one winter evening—six persons riding inside the carriage with the windows closed, and one on the box-seat. Within three days all those who had ridden inside the carriage had been attacked by the influenza, while the one who sat outside escaped. The question arises, did those who suffered receive infection from one of their number, or were they simply more liable to the onset of the disease because their vital forces were lowered at the moment by the inhalation of foul air?

None of these things can be regarded as settled by the report. Advocates of either the infectious or the atmospheric theory may regard the cases we have quoted as supporting their opinions; it is difficult to say to which side the balance of evidence inclines. An equal amount of doubt seems to hang over the question of immunity. Does one attack preclude another? If that were certain we should all be tempted to protect ourselves by inoculation with a mild form of the disease. But the majority of the medical men quoted seem to think that not only does one attack not prevent another, but that it even predisposes to it. The medical officer of Ulverston, who had suffered from influenza in 1857, took it in 1890, and had three attacks in 1891. Dr. Davidson, of Congleton, quotes the case of an unfortunate lady who having just recovered from influenza at Congleton went to London where the the epidemic was more recent. There she fell ill again; and on her recovery she went to Scarborough. At Scarborough the epidemic had just broken out, and the lady fell ill again—a third attack within a period of six weeks. But doctors proverbially differ. Dr. Caldwell Smith, of Motherwell, says that of the cases he attended in November and December, 1891, and January, 1892, 75 per cent. had escaped the two previous epidemics. With regard to the 25 per cent. in whom the disease proved recurrent he divides them into four classes: Those suffering from some chronic lung affection, such as bronchitis and phthisis; those in whom the previous attack had left some permanent mischief, such as endocardial affections; those who suffered from some organic disease, and had little resisting power; and about 15 per cent. in whom he could diagnose nothing predisposing to a second attack. His opinion is, therefore, that for the great majority of people one attack confers immunity from another for some time, though he does not venture to fix a numerical limit. One example of immunity which Dr. Caldwell Smith quotes is certainly curious enough. It is well known that simultaneously with the epidemic in the human race there was a wide spread of influenza, known in stable language as "pink-eye" in horses. At a posting establishment in Motherwell several horses were attacked, and two grooms were specially set apart to attend them. The other grooms in the yard all took influenza; the two who looked after the sick horses alone escaped.

Dr. Parsons, who writes this portion of the report, seems himself to incline to the theory that one attack confers a certain amount of immunity. He took the trouble to test it by the statistics of large institutions, and those he gives of the industrial schools at Swinton, near Manchester, are curious and instructive. These schools suffered severely in the first epidemic (1889-90), as many as 171 out of 589 children having been attacked, or 29 per cent. In the first epidemic of 1891 influenza broke out again, but in a milder form, there being only 35 cases. Of these four had had the disease the previous year, and now again suffered from it. But of the children at the schools in 1891 as many as 449 had been present during the previous epidemic, of whom 150 had suffered from it. This gives 2·6 per cent. as the number in whom one attack confers no immunity from a second. Of the 299 who escaped influenza in 1890, 17, equal to 5·7 per cent., were infected now. The inference is that one attack gives an immunity which at the end of a year diminishes the liability to another by at least one-half.

It seems possible that whole districts may by one epidemic acquire at least partial immunity from another. London as a whole suffered severely in all the three epidemics, but we have no certain information as to the proportions in which the various districts suffered each time. A few facts on this point would have been of great value. The course of the epidemic seems at least to have varied on each occasion, and many places suffered severely in one and only slightly in another. It cannot be said that the topography of a place seemed to alter its chance of infection. In fact, the contradictory statements made by different observers of equal honesty make their evidence almost wholly valueless. In the single county of Lincoln we find three wholly different opinions. Drs. Barton and Eminson think the disease was most prevalent in high and exposed places such as the Wolds; the medical officer for the Stamford district says it was more severe in low lying districts than on the higher ground; while the medical officers of Spilsby and Sleaford found that topography make no difference, the disease being as prevalent in the fens as on the high ground, and vice versa. This seems at least to go against the atmospheric theory, which implies atmospheric conditions favourable or the reverse to the conveyance of the disease.

Nor, though influenza is, on the whole, a winter disease, does it seem to be aggravated by those conditions of cold and exposure to which the poor are most liable. "In London the deaths from influenza," says Dr. Parsons, "were most numerous, relatively to the population, in the sanitary areas of St. George's (Hanover Square), Kensington, Chelsea, and Lewisham, quarters containing a large proportion of good houses and wealthy or well-to-do people; the proportion was also above the average in St. Giles, City of London, Whitechapel, Rotherhithe, Paddington, Wandsworth, Battersea, and Plumstead districts, differing much one from another in character. The proportion was lowest in St. George's (Southwark), Westminster, Woolwich, Poplar, Shoreditch, and Newington districts, for the most part inhabited by the working classes. Thus neither riches nor poverty seems to give protection. If one may draw any inference at all, it is that the best security comes from the hardy constitution and well braced frame that belong to manual labour.

